# Origins of CD8 tissue resident memory

**DOI:** 10.3389/fimmu.2026.1834618

**Published:** 2026-06-11

**Authors:** David J. Topham

**Affiliations:** Department of Microbiology and Immunology, School of Medicine and Dentistry, University of Rochester, Rochester, NY, United States

**Keywords:** CD8, infection - immunology, memory, resident, T cell

## Abstract

The concept that immune cells, specifically T cells, become established and reside in peripheral tissues, especially at barrier sites, has been around for several decades. Recent studies have revealed substantial heterogeneity at the cellular level that depends on the type of immune response and tissue environment, among many other factors. Nevertheless, some features are common to most resident memory T cells, especially CD8+ cells. These include certain surface receptors and transcription factors. It is important to understand how tissue-resident memory (T_RM_) T cells develop and function, as these cells are critical for subsequent protective immune responses when the same or antigenically drifted variants of a pathogen are encountered. CD8+ T cells resembling T_RM_ can be found in solid tumors and correlate with prognosis. It is attractive to consider devising strategies to generate T_RM_ for preventive or therapeutic interventions. While there is information on how resident memory T cells develop, our understanding is incomplete, and many questions remain. Here, we review the origins and early observations on the concept of memory T cell residency, models of how and where resident memory forms, and the open questions that remain.

## The way we were (memory)

Learning is a crucial part of development, enabling us to adapt to our world. Few appreciate that we have (at least) two major organ systems with the ability to learn and remember. Just as we know and use facts and experiences to survive in society, our bodies experience, learn, and remember things that disrupt the homeostasis of our tissues. Memory is a term we use to describe the ability of our minds to recall past experiences. Networks of long-lived neurons and synapses record our experiences and emotions. Memory also applies to our immune system’s ability to recall past encounters with benign and malignant agents originating from inside and outside our bodies. In the immune system, networks of long-lived lymphoid and myeloid cells develop to form immune memory. While the central nervous system is anatomically restricted, the immune system is distributed throughout the body. The immune system is sophisticated enough to know that some tissues, such as the barriers to the outside world (skin, digestive tract, and airways), are more likely to repeatedly encounter the things it has learned about. Consequently, it develops strong immune memories in these tissues to regulate and protect us. These memories are carried by immune cells, with the most effective known as memory B cells and T cells. These cells either produce antibodies (B) or regulate, help, and eliminate damage and infection (T). Because these cells are crucial for protecting and preserving the integrity of our bodies, it is essential that we understand how they form and are maintained. Here, we will discuss memory T cells that are predominantly located in non-lymphoid tissues.

## You take the high road, I’ll take the low road (pathways and routes)

The idea that T cells differentiate into long-lived memory subsets with different probabilities of circulating in specific circuits around the body or remaining resident in a single tissue without circulating has been around for decades. In 1959, James Gowans demonstrated that cells collected from the thoracic duct, where lymphatic fluid and cells are returned to the blood, were qualitatively different from cells collected from the blood or lymphatic tissues (reviewed in Nature Milestones: T cells (1). The idea formed that immune cells could circulate through distinct circuits. The observations suggested that some immune cells leave the blood, enter peripheral tissues, and then drain back into circulation through the lymphatic system. Later studies in mice and humans indicated that at least two broad subsets of T cells could be distinguished based on whether they were likely to recirculate from blood into secondary lymphoid organs (SLOs) and back, or into peripheral non-lymphoid tissues (NLTs), later returning to blood via lymphatic drainage. These subsets were termed Central Memory T cells (T_CM_) and Effector Memory T cells (T_EM_) distinguished by expression of specific adhesion molecules and chemokine receptors (2, 3).

## Déja vu (sensing that we’ve experienced something before)

While the topic of “tissue-resident memory” began to take hold in the early 2000’s (4–7), concepts of resident memory cells and strong evidence that T cells could take up long-term residence in NLT without recirculating existed earlier. Studies by Hickey observed that effector T cells were competent to enter the central nervous system (CNS), and memory cells within the CNS did not readily exit back into circulation (8). In 1996, work in the Davis lab reported the development of quadrivalent major histocompatibility complex (MHC) reagents loaded with specific antigenic peptides for use in fluorescent flow cytometry (9). These MHC tetramers are used to stain antigen-specific T cells for flow cytometric analysis and sorting, allowing for deeper characterization (phenotyping) and enumeration of antigen-specific memory and activated T cells. In 2001, Dr. David Masopust, working with Dr. Leo LeFrancois, demonstrated that after systemic infection with *Listeria monocytogenes*, specific tetramer-positive CD8 T cells could be detected at elevated frequencies in nearly every tissue sampled, even long after the infection had been cleared (10). Around the same time, using whole-body cryosections of mice, the Jenkins lab demonstrated the presence of antigen-specific CD4+ memory T cells in every tissue after intravenous (systemic) immunization with specific peptide plus lipopolysaccharide (11). These observations clearly showed that memory CD4 and CD8 T cells distribute throughout the body after infection and immunization. However, the issues of residency and recirculation had not yet been assessed in a similar context. T cells in peripheral tissues have cell surface phenotypes that closely resemble the circulating T_EM_ cells identified by Sallusto and Lazavecchia, which was consistent with the idea that certain memory T cell subsets were more likely to infiltrate NLT, partly because they had gained receptors to enter peripheral sites, and lost expression of receptors that direct them into lymphoid tissues. However, phenotypic discrepancies between memory T cells from different tissues and studies of T cell-mediated secondary immunity indicated that the immune surveillance and protection involved more than just circulating and lymphoid immune cells. In the lung, Gerhard demonstrated in an influenza model that heterosubtypic immunity (protection against a related but serologically distinct virus) was long-lived and required CD4 and CD8 T cells (12). Woodland et al. then demonstrated, using a parainfluenza virus model, that virus-specific CD8 T cells declined in number over time in the lung (13). The critical concept is that secondary protection is determined by the location and number of T cells in peripheral tissues, rather than by the total number of T cells in the organism. It was then demonstrated that, after memory had been established, the decline in cell numbers in the tissue could be accelerated by inhibiting the integrin CD49a (4). Inhibiting or deleting CD49a appeared to redistribute influenza-specific CD8 T cells from the periphery to the SLO, yet abrogated secondary immunity. An increased number of memory T cells in the SLO did not compensate for the time it takes to recall cells to the lung (4). The idea was put forward that there were at least *“two populations of … memory T cells, one defined by expression of VLA-1 [CD49a] that is resident or passing relatively slowly out of the tissue, and another more rapidly recirculating pool”* (4). This was the first study to identify a functional marker of T_RM_. At about the same time, using parabiosis, seminal studies showed that non-recirculating memory T cells are a feature of multiple peripheral non-lymphoid tissues (7). Parabiosis involves surgically connecting a donor and a host mouse, which typically differ in a congenic marker (CD45 or Thy 1) expressed on T cells. The donor mouse is primed, usually by infection, to generate both circulating and resident memory T cells. After a period of conjoinment, tissues from the host animal are sampled for the presence of donor-origin memory T cells. Circulating cells equilibrate between the host and donor mice, while resident cells do not (or are substantially biased in distribution). To this day, parabiosis experiments remain the gold standard for demonstrating tissue residency. Later, in 2009, a study from the Carbone lab demonstrated that memory CD8 T cells could form and persist in the skin for long periods after infection, were critical for rapid, local immunity against reinfection, and a majority (85%) expressed CD49a, distinguishing them from circulating memory T cells (14). These early findings built the foundation for the formal definition and characterization of T_RM_ cells as a distinct T cell lineage that permanently resides in tissues, separate from the traditionally defined circulating memory T cell subsets. They also established the concept that secondary T cell immunity was linked to the frequency and number of T cells in the organ. This “closed the loop” on the paradigm that there is a widespread diaspora (15) of memory T cells into peripheral tissues following an infection, which subsequently become resident.

## Crouching tiger, hidden dragon (recognizing residency)

In the literature, the terms “residency, lack of recirculation, and tissue retention” are used interchangeably. In most cases, they reference observations that some memory T cells appear to enter a tissue and stay there. This outcome reflects a constellation of biological functions that stabilize a cell population within some tissues. These functions are not passive or a default pathway. For instance, CD69 directly interacts with the TM4 (transmembrane helix 4) domain of S1P1 (16). It acts as an agonist that induces S1P Receptor (S1PR) internalization and degradation, thereby suppressing S1P1 signaling, which T cells need for efficient migration towards draining lymphatic vessels. “Residency” typically refers to cell populations that appear to remain stable in number over time, akin to a permanent address. “Lack of recirculation” can only be observed in the context of fate-mapping or parabiosis experiments, in which the cell populations being tracked don’t appear in the blood, a different tissue, or the tissue of an adjacent parabiont. “Retention” is a term that implies an active process, including migration arrest, stasis, or adhesion. The function of CD69 fits this category. In earlier days, we often referred to CD49a as the “retaining” factor for T_RM_ in the lung (4), presumably by mediating adherence to collagen IV (17, 18), but retention is a misnomer. Although it seems to promote residency, it was discovered that CD49a on memory T cells functions in cell locomotion and motility in the tissue (19), which at first seems counterintuitive. For reasons such as this, the terms we apply often matter because they can color one’s thinking.

Residency and retention do not mean quiescent. It is essential to recognize that immune cells, particularly T cells, are dynamic. Few, if any, immune cells are truly sessile. The concepts of “residency, non-recirculating, and retention” imply a certain lack of movement. But that could not be further from the truth for memory T cells, even T_RM_. They are never truly at rest. T cells function by sensing the tissue microenvironment (20). They are always on the move, responding to chemical and physical cues that regulate motility, direction, activation, inhibition, antigen presentation, adhesion, survival, and growth. Motility is required for surveillance, and live imaging of T_RM_ has shown them to be constantly moving in the tissue, though at slow speeds and limited distances (19, 21, 22). Although the distribution of T cells among different tissues has a stochastic element, there are clearly features that affect the likelihood that cells will enter one tissue or another, such as active inflammation and receptors for chemokines and adhesion molecules.

The possibility of entering a particular tissue is then determined by the constellation of adhesion receptors and chemical signals expressed by the T cells as well as by the tissue itself, beginning at the tissue vasculature. All T cells can travel in the blood. They roll along the walls of the vasculature, sensing the vessel walls. Naïve T cells are more likely to enter lymphatic organs because they have receptors (CD62L, CCR7) that match ligands (sialyl Lewis X glycans and CCL19/CCL21) on the vasculature of those tissues. Activated and memory T cells expressing LFA-1 and CD49d (VLA-4), for example, can enter any tissue, but are more likely to enter an inflamed tissue because their ligands (ICAM-1 and VCAM-1) are upregulated in the vasculature at sites of inflammation, as is the local concentration of chemokines trapped by the vascular glycocalyx (23, 24). Antigen is not required for entry into inflamed or healthy sites, although it does promote accumulation (25, 26). Tissue-specific homing occurs in some tissues. T cells are educated during priming to express additional adhesion, addressin, and chemokine receptors that increase their likelihood of entering specific tissues. For example, dendritic cells in the skin can induce T cells to express Cutaneous Lymphocyte Antigen (CLA), which binds to E-selectin expressed by the inflamed skin vasculature (27, 28). The skin-homing T cells also express CCR4 and CCR10, whose expression is promoted by vitamin D production, to aid exit and positioning in the skin (28, 29). In turn, retinoic acid (a Vitamin A analog) produced in the intestine instructs DCs to direct T cells to express CCR9 and integrin α4β7 (for Mucosal Addressin Cell Adhesion Molecule, MAdCAM), which directs T cells to exit the circulation into the gut (30).

Collectively, these relationships underscore the importance of understanding the function of the so-called “markers” used to identify T cell subsets (31). For T_RM_, cell-surface expression of the markers CD69 and CD103 is commonly used in the literature. CD69 is associated with T cell activation, but its primary function is to inhibit the S1P1 receptor (S1P1R). S1P gradients guide T cells out of lymph nodes and peripheral tissues and into the lymphatics (32–34). Reducing the ability to sense this exit cue promotes retention at the site. For T_RM_, it is less clear whether CD69 is triggered by TCR-mediated recognition of antigen or by some other signals. On the other hand, CD103 (in a heterodimer with β7 integrin) binds its ligand E-cadherin, which is expressed at cell-cell junctions, especially in epithelial cells (35). CD103 was identified on mucosal tissue T cells and associated with their compartmentalization (36, 37). It was first shown to be expressed on skin-resident memory CD8 T cells in a model of Herpes Simplex Virus (HSV) infection (14). CD103 may also promote cytotoxic CD8 T cells’ function in some tissues, since it was shown to be necessary for T cell-mediated renal allograft rejection (38, 39). ItgaE (CD103/αE)-deficient memory T cells do not accumulate efficiently in peripheral tissues, resulting in a decrease in T_RM_ numbers, which suggests that αE is important for optimal T_RM_ establishment (40–42). Antagonizing αE increased cell velocities in a live imaging study, which is consistent with a function of tethering the cells to the tissue itself (19). It is less clear whether CD103 is necessary to maintain residency once T_RM_ become established in the tissue, as its inhibition results in a gradual rather than sudden loss of resident memory (42, 43). CD49a pairs with the integrin β1 to form an adhesion heterodimer known as VLA-1 (44–46). VLA-1 binds to collagens I and IV in the extracellular matrix. It is the only integrin ligand for collagen IV, which is unique among collagens in its mesh-like (non-fibrillar) sheet structure located at the base of epithelial and endothelial cell layers (18, 47). CD49a does not appear to play a role in T cell migration to, or accumulation in, tissues (48) and instead seems to function in retaining the cells (4, 31, 48, 49). In an influenza infection model, experiments blocking CD49a with antibodies after memory had been established, or with T cells genetically deficient for CD49a, reduced the number and frequency of virus-specific CD8 T cells in the lungs (4). Itga1 genetic deficiency also reduced CD8 memory T cell retention in the skin after herpes simplex virus (HSV) infection (48). Also in the skin, vaccination to generate local virus-specific CD103+ CD8 T cells subsequently cleared a herpes simplex virus skin challenge more effectively than controls (21). Many other vaccination and challenge studies in a variety of tissues (reproductive tract, skin, and lung) have demonstrated diminished protection by tissue-localized memory T cells when their development is inhibited.

Other receptors associated with residency, although not exclusively, include CXCR3, CXCR6, and CD101 (50, 51). Chemokine receptor signals are crucial for guiding T cells into and within the tissues, but not for tissue residency per se. They are important for positioning cells within the tissue structure and microenvironment. CD101 inhibits T cell proliferation through blocking IL2 production and signaling (52), perhaps limiting inappropriate T_RM_ reactivation. However, the capacity to become tissue-resident is not determined by any single receptor or transcription factor. Residency requires a constellation of adhesion and chemokine receptor up- and down-regulation. The mere presence or absence of any of these markers, including CD49a, CD69, or CD103, is insufficient to classify CD8+ T cells as tissue resident (33, 53–56).

## Of mice and men

Much of our understanding of memory T cell localization in tissues is derived from using mouse models. Obvious reasons are that mice are both readily available and provide relatively easy access to virtually any tissue site. Human peripheral tissues and SLO are much more difficult to sample. Most studies rely on cadaveric and biopsy material, though results from a wide range of tissues have been reported, including skin, female reproductive tract, lung, digestive tract, brain, kidney, and tumors (37, 57–59). Fortunately, many cell-surface phenotypes and markers of probable T_RM_ are similar to those in mice, including CD49a, CD62L, CD69, CD103, CD101, and S1PR1 (60). Parabiosis is obviously off the table, but information has been learned from tissue transplants (61). As for transcriptional regulation, there are both similarities and differences. There is no human homolog for Hobit, but upregulated expression of RUNX3, Itga1, and Itgae, and downregulation of Eomes, Tbet, and Klf2 are conserved between human and mouse (58). The expression of Inducible CO-Stimulator (ICOS), a receptor that inhibits Klf2 and has been shown to cluster T_RM_ populations, is also conserved (56). The importance of TGFβ signaling in regulating T_RM_ development and integrin expression is well established in both species. Also, like mouse T_RM_, cross-presenting dendritic cells (DC) have been shown to drive human T_RM_ development (62). It seems likely that T_RM_ development, regulatory genes, and phenotypes in humans are at least roughly analogous to those in mice.

## A mosaic

According to recent single-cell data, memory CD8 T cells with tissue-resident potential are heterogeneous (56). Just as T cell migration is determined by a constellation of features, memory T cells integrate a constellation of inputs, including location, stimulation history, antigen, and environment (20), that produce a mosaic of cell phenotypes (56). The cells frequently reflect the tissue they are in. There is no one set of characteristics that defines a resident cell, suggesting T_RM_ remain highly adaptable to their environment after initially forming. If cell surface markers are to be used, the most reliable and least ambiguous combination (at least for mice) is CD62L^neg^, CD49a+, CD69+, and CD44+ (56). Neither CD69 alone nor in combination with CD103 was found to be satisfactory, despite their common use to define T_RM_. Although CD49a was recognized early as a feature of some T_RM_ (4), the lack of commercially available antibodies for flow cytometry initially limited its widespread adoption for phenotyping cells. Fortunately, this limitation was solved some time ago. In an influenza model, the CD49a+ CD103+ and CD49a+ CD103– cells account for 65-90% of the virus-specific CD8 T cells in the tissue, depending on the antigen (19). After selecting cells that are CD62L negative and express CD69 and CD44, at least 4 functionally and phenotypically distinct populations of memory CD8 T cells were identified, with those expressing CD49a demonstrating the highest functionality (63). There is a CD49a^neg^ CD103+ population that appears short-lived and functionally limited (19, 63). Many naïve T_RM_ express CD103, and some recently migrated T cells and naïve human T cells express CD69. However, there are no T_RM_ that express CD69 and CD103 but do not express CD49a. On the other hand, a high proportion of long-term memory CD8 T cells in the lung express CD49a and, of those, many also express CD103. Experiments that use CD103 alone, with or without CD69, risk excluding many likely resident memory T cells that express only CD49a. The remaining integrin-negative T cells form a mixed population that includes some with an effector memory phenotype, as well as a minority of cells of ambiguous lineages, which may consist of resident cells with diverse phenotypes. These observations suggest that, at least for the lung (and likely the skin and gut), the majority of T_RM_ express CD49a, CD103, or both. To complicate things further, memory T cell phenotypes exhibit plasticity, and it is unclear how interchangeable the different subsets are.

Memory CD8+ T cells that express CD69, CD49a, and CD103 play protective roles in a wide range of tissues, both during infection and cancer. In clinical studies, the expression of CD49a and/or CD103 by tumor-infiltrating lymphocytes (TILs) is associated with more favorable outcomes (64–66). However, it remains to be determined whether integrin expression by TILs is a property of tumor residency or is associated with other capabilities. It would make sense, for example, that CD49a+ TIL could be more efficient in tumor surveillance due to their increased motility; however, it has been proposed that collagen binding could sequester TIL away from tumor cells (67), suggesting the tumor microenvironment needs to be considered. The fact that these integrins are induced in models of both infection and cancer suggests the signals leading to their development are common to both diseases. Given the significance of resident memory T cells in infection and cancer, several efforts have been made to determine their origins and developmental pathways. However, the evidence is incomplete at best and tenuous.

## Dad, where do T_RM_ come from?

Studies suggest that some T cells are predisposed to tissue residency, with programming beginning while they are still in a naïve or pre-activation state. A substantial proportion of naïve CD8 T cells in the periphery express CD103, for example. CD8 T cells begin expressing CD103 in the thymus upon transition from the CD4+ CD8+ double positive to CD4– CD8+ single positive stage (68, 69). As a result, some naïve CD8 T cells in the periphery that express CD103 may be recent thymic emigrants (69). However, since not all naïve CD8 T cells are recent thymic emigrants or express CD103, many must lose CD103 expression before activation. While all CD8+ T cells may be CD103+ at some developmental stage, which may affect chromatin accessibility, it’s not clear which of these will become T_RM_. For the T cells that do develop into memory, our data and those of several other labs indicate that migration into peripheral tissues drives stable CD103 expression by memory T cells. Like many activation markers, CD69 expression is dynamic and triggered by recent antigen encounters, TCR signaling, and recent tissue entry (32, 33). Activated T cells in the LN initially express CD69 and then lose expression before exiting, only to regain it after antigen encounter in the peripheral tissues (33, 70). This dynamic regulation is consistent with the role CD69 plays in regulating signals through the S1P1 receptor for lymphatic homing and tissue retention (33). The mechanism by which CD69 expression is maintained by late T_RM_, for instance, whether it remains expressed in response to an antigen, is yet unclear (34, 71, 72). CD49a, which was initially named Very Late Antigen-1 (VLA-1) after *in vitro* activation studies (73, 74), is not very late at all, and is actually expressed early *in vivo*, though still not driven by activation alone. In our hands, it is expressed on virus-specific CD8 T cells as soon as they reach detectable frequencies during primary influenza infection (4). It has not been formally determined that these cells are T_RM_ precursors. Other stable markers that are less T_RM_-specific, such as CCR7, CD44, and CD62L (L-selectin), are up- or down-regulated, respectively, on activated T cells in the T_EM_ and T_CM_ subsets (75). It seems, then, that there is no reliable way to identify intact T_RM_ precursor cells at present.

Despite the interest and practicality of having markers to reliably identify T_RM_ and T_RM_ precursors, recent single-cell gene expression data reveal remarkable heterogeneity in populations of resident memory T cells, which can also vary depending on the tissue from which they were isolated (56). Nevertheless, it was noted that the downregulation of CD62L, upregulation of CD44, and expression of CD69 and/or CD49a may be the most reliable features of T_RM_ (56). CD103 is a more ambiguous feature of T_RM_. This raises questions about conclusions drawn from studies that used any single marker to identify and characterize T_RM_. It is unclear whether studies using CD103 alone are biased by the fact that many of the cells were part of the short-lived, functionally limited CD103 single-positive population, and how many were mixed with integrin double-positive (CD49a+ CD103+) memory T cells, thereby blurring functional and gene-expression distinctions. Such distinctions based on integrin expression may require further evaluation, and conclusions drawn from limited marker expression may need careful re-examination. One attractive approach to sorting out (pun intended) these issues would be to apply lineage-tracing tools to track integrin-expressing T cells during activation and memory development. Combined with conditional deletion, these could shed light on the functions and timing of transcription factors and integrin expression in acute, memory, and chronic T cell responses.

## One if by land, two if by sea

Several cytokines have been implicated in the expression of the integrins CD49a and CD103, as well as the development of T_RM_ (43, 48, 53, 76, 77). These include IL-15, IL-12, and TGF-β (48, 53, 76, 78, 79). The picture is far from clear, though. IL-15 promotes proliferation and survival in some memory and resident memory T cells but is not universally required (80). TGF-β triggers the partial downregulation of T-bet and complete shutdown of eomesodermin (Eomes) expression (43, 81–83). The low-level *T-bet* expression is sufficient to induce IL-15R, whose signaling can drive *Hobit*, another (mouse) T_RM_ signature transcription factor (81, 84). *In vitro* activation of CD8 T cells in the presence of TGF-β leads to the expression of CD103 and CD49a (48) and has been used to generate T_RM_-like T cells (48, 85). Low glucose and high fatty acid concentrations in the media perpetuate this response (85–87). TGF-β receptor deficiency leads to a significant reduction in memory T cells in NLT (88). While the addition of IL-12 alone or in combination with TGF-β enhances CD49a expression, it prevents or inhibits CD103 (48). Certainly, the existence of CD49a+ CD103– integrin single-positive and CD49a+ CD103+ double-positive T_RM_ *in vivo* (63), and the observation that CD49a and CD103 expression are independently regulated (48), suggest that there are diverse paths for T_RM_ differentiation. Unfortunately, none of these *in vitro* models accurately reflects the myriad of signals provided to T cells in tissues during memory and effector T cell differentiation, and they leave much to be desired.

## Sagittarius 6EQUJ5 wow! (where signals come from)

Where do the signals that drive T_RM_ generation come from? Two models have been proposed and are supported by experimental data. They are sometimes portrayed as either/or, though they are entirely compatible with one another, and both may be correct. One is called the “local divergence” model, and it proposes that T_RM_ receive signals and develop in peripheral tissue sites where TGF-β activation and signals are provided by the local environment (84, 89–91). The second is a “systemic divergence” model, in which T cells destined to become T_RM_ receive signals during priming (or earlier) in the SLO, including the spleen, that predispose them to a T_RM_ fate (84, 92, 93). While there is evidence supporting both models, there are significant issues with each, not least the need for reconciliation. Fortunately, they are not mutually exclusive. They are also limited, like many studies, because they have been based on incomplete or flawed conventions for identifying T_RM_ cells.

## Discrete and continuous models of T_RM_ development

**Figure 1:**
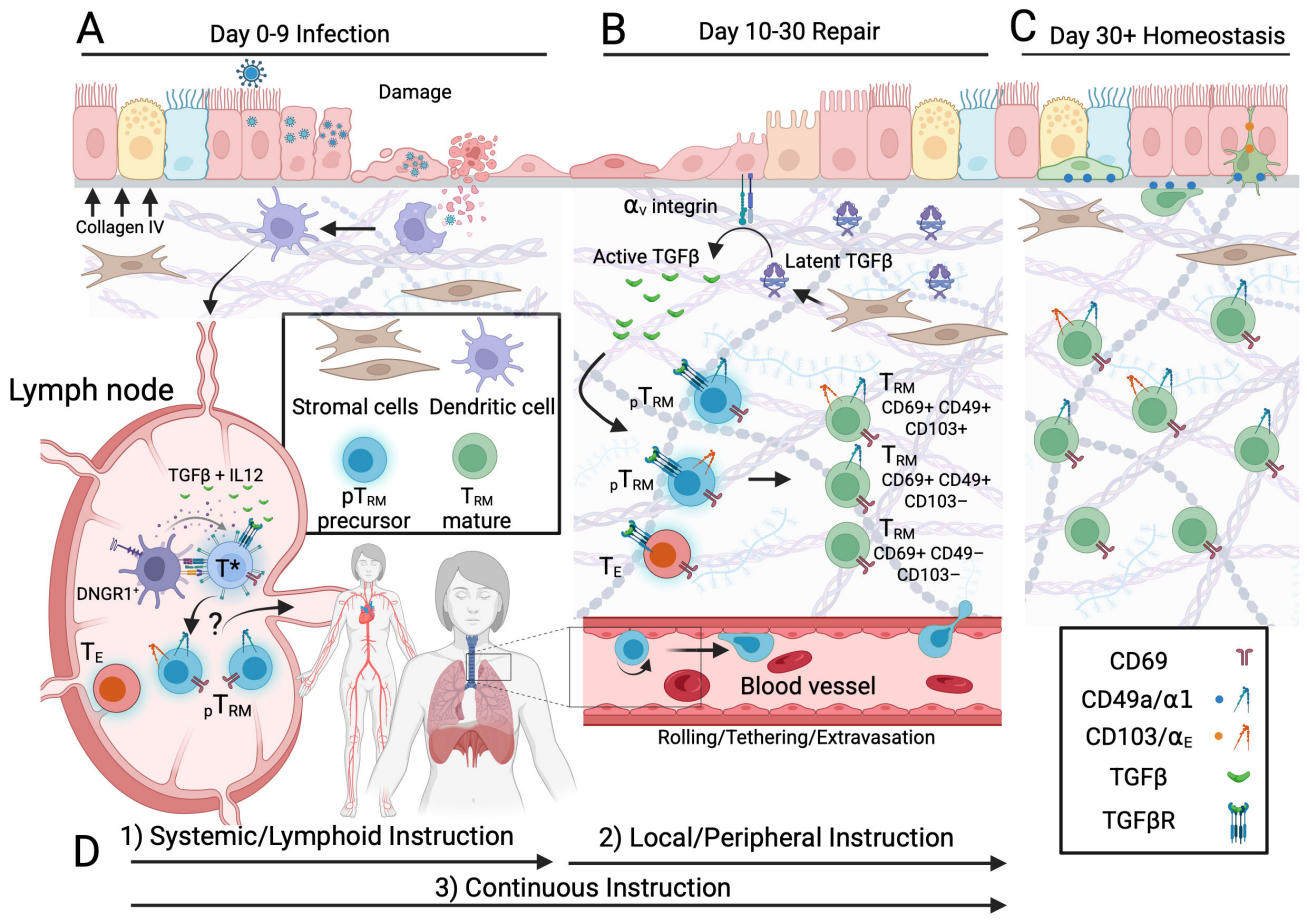
Continuous and discrete tissue resident memory CD8+ T cell development during primary infection in the lung epithelium Virus infection of the lung epithelium induces innate immune responses and damage. **(A)** Dendritic cells phagocytose damaged and dying cells, then carry antigens to the draining lymph nodes and the spleen. Specialized populations of DC that express DNGR1 (CLEC9) (in mice) or CD1c (human) cross-present antigen to naïve CD8+ T cells that become activated and express T_RM_-associated genes. DCs express TGF-β in both secreted and cell surface forms. Some express αV integrin that can activate TGF-β, and some may also secrete IL-12. Many activated T cells begin to express CD49a, and some may be precursors of resident memory T cells (pT_RM_). **(B)** Activated effector and memory precursor CD8+ T cells leave the lymphatic organs and travel through the circulation to blood vessels in the infected tissue, as well as healthy tissues. After extravasation, they patrol the tissue for infected and antigen-bearing cells. During the repair phase after infection, αV integrin-expressing cells (dividing epithelial cells in airways and keratinocytes in skin) activate latent TGF-β (secreted by stromal cells) bound to Latency-Associated Peptide. KLRG1 negative effector and memory precursors receive TGF-β signals and further differentiate into CD69, CD49a, and CD103 expressing T_RM_ subsets, as well as (presumably) integrin-negative subsets. **(C)** After several weeks, the tissue has been repaired, and resident memory T cells patrol the tissue for weeks or months, positioning in proximity to ligands such as collagens I (interstitial) and IV (subepithelial) and E-cadherin (epithelial), depending on both chemokine signals and adhesion molecule expression. **(D)** According to current models, signals to differentiate (TGF-β IL12) into resident memory T cells may be delivered (1) only in the lymph nodes, (2) only in the tissue, or (3) continuing from the lymph nodes to the tissue, possibly accounting for heterogeneity in the memory T cell pool. Created in .

Here, we propose reconciling these into a model in which signals for residency potential are continuous, beginning in SLO and progressing in peripheral tissues, finally forming mature T_RM_ cells (**Figure 1**). This model incorporates T cells receiving instruction from specialized DCs at the naïve stage during initial activation (**Figure 1A**). These instructions include TGF-β and may also include cues (e.g., retinoic acid, vitamin D) that trigger the expression of tissue-specific homing receptors. Tissue-specific homing via lymph node imprinting and the expression of specific chemokine and adhesion receptors is limited to barrier sites, such as the skin and gut (28–30, 94, 95). Other peripheral tissue lack such defined cues. After activation, proliferation, and differentiation into KLRG1-negative effectors, they exit the SLO, briefly enter circulation, and migrate to inflamed and some healthy tissues (**Figure 1B**). In infected or damaged tissues, activation of latent TGF-β, augmented by expression of αV-integrins during tissue repair, would drive further local differentiation (**Figure 1B**). Signals (including IFN-β, IL-15, TNF-α, IL-12) from the local microenvironment promote further survival and differentiation into mature resident cells (**Figure 1C**). The T cells that receive instructions in the SLO, but end up in healthy tissues could still become resident, but might not express CD103, accounting for some of the observed heterogeneity. Likewise, KLRG1– effectors that didn’t receive residency cues in the SLO, could still enter peripheral tissues under repair and receive local cues to become resident, further adding to the heterogeneity. We discuss the evidence and implications for these discrete and continuous models below.

## Persistent signals that reinforce differentiation and survival

Elegant experiments demonstrate a vital role for specialized dendritic cells in LNs, as inhibition of these cells reduces, but does not abrogate, the number of T_RM_ in tissues. Migratory DC expressing cell surface TGF-β may predispose T cells to a T_RM_ fate, in an apparently non-antigen-specific manner (96). Deficient cross-presentation by DC resulted in a threefold reduction in the number of dermal T_RM_ cells after vaccinia virus infection (93). In mice, during vaccinia infection of the skin, antigen cross-presentation by BATF3+ DNGR-1+ (CLEC9) DC was required for optimal T_RM_ cell priming (92, 93). DNGR-1^+^ DCs promoted T_RM_-associated transcription-factor induction by CD8^+^ T cells in the lymph nodes (LNs), dependent on the provision of cytokines including TGF-β, IL-12, and IL-15 (92, 93). The same paper also showed that inhibition of DNGR-1 limited T_RM_ cell generation in the lung and, consequently, secondary immunity in an influenza infection model. In this model, naïve CD8 T cells are primed by antigen cross-presented by DNGR-1+ DC bearing surface TGF-β (93). In humans, a population of lung-tissue-resident CD1c+ (not to be confused with cDC1) DCs that express TGF-β on their cell surface can drive CD103 expression on CD8 T cells and promote CD8 T cell accumulation in lung epithelia, both *in vitro* and *in vivo* (62). The significance of these observations is that they show optimal T_RM_ generation depends partly on signals delivered by specialized DC in the LN. Note that in both the skin and the lung, T_RM_ generation was not completely abrogated by inhibiting DCs, but rather reduced, leaving open the possibility that T_RM_ differentiation continues in the tissues or occurs *de novo*.

Several studies of T cells in various tissues, including gut, skin, lung, and brain, highlight the importance of tissue-derived TGF-β signals in driving integrin expression and T_RM_ differentiation. TGF-β is secreted in an inactive state, bound to latency-associated protein (LAP) (97). Activation requires release from LAP and can be mediated by αV-integrin binding to the TGF-β–LAP complex (98). αV-integrins are expressed by several cell types, including migratory DC (96, 97), keratinocytes performing tissue repair in the skin, and keratin-5 (KRT5) positive epithelial cells in the lung (91, 99, 100). Takamura proposed that the areas of tissue repair following influenza infection form “niches” where T_RM_ develop, and called these Repair Associated Memory Depots (RAMD) (91, 101). In these niches, TGF-β is activated by dividing αV+ epithelial cells, providing a rich local microenvironment that promotes T_RM_ differentiation. TGF-β is in every tissue, though the existence of T_RM_ niches in other tissues remains to be demonstrated. Regardless, the evidence of TGF-β’s important role in other tissues supports the local divergence model of T_RM_ development. What appears to be lacking are clear links or distinctions between the “T_RM_-precursors” primed by LN DCs and the mature T_RM_ derived from these precursors, as opposed to those that may develop entirely from local signals. Understanding these lineages may explain the heterogeneity in T_RM_.

There may be other influences on T_RM_ diversity. Several lines of evidence point to a biased selection of CD8 T cells destined to become T_RM_. An elegant fate-mapping study using adoptively transferred, retrovirally barcoded, OT-I thymocytes demonstrated an unequal distribution of clones between the LN and the skin, suggesting a selective process (102). Although that study used fixed-TCR transgenics, TCR affinity and duration of antigen signaling can influence memory T cell fates (84, 103, 104). T_RM_ formation is influenced by TCR/pMHC affinity, although the results are inconsistent. Memory T cell differentiation can be favored by low-affinity TCRs and longer time in the LN (93, 104). Blockade of T cell egress from LN with FTY720 increases T_RM_ generation, at least when LN differentiation is inhibited (93). On the other hand, in the kidney and brain, T_RM_ had higher-affinity TCRs, which were interpreted as possibly favoring those T cells that can sense the lowest levels of antigen and are the least likely to be promiscuous, features that would be useful in a tissue where T cell activation can lead to dire outcomes (105). It has also been shown that the most expanded clones are less likely to persist as long-term memory, although even some KLRG1+ T cells retain some capacity to become resident later (106, 107). These observations of biased selection generally support a central differentiation model.

Another unresolved issue in the resident memory literature is the extent to which T_RM_ are tissue-specific or infection-limited in their distribution. Of course, the presence of T cells with a T_RM_ phenotype in tumors shows that they are not limited to infection alone. T_RM_ have been found in virtually all peripheral tissues. There are, however, significant differences in cell phenotypes that appear tissue-specific and possibly tissue-directed, at least with respect to surface phenotypes. Many studies of T_RM_ utilize tissue-restricted infections (e.g., HSV, influenza, vaccinia) or tumor models. Early observations by LeFrancois and Masopust demonstrated the widespread distribution of antigen-specific CD8 T cells to multiple tissues using *Listeria* and LCMV models of infection, both of which are systemic (10, 108), making it difficult to separate whether infection or antigen in the tissue is required to establish local memory. In an influenza model in which the infection is strictly limited to the airways, we found widespread distribution of flu-specific (NP tetramer-positive) CD8+ T cells in multiple tissues months after infection (4). A similar phenomenon was observed in which T_RM_ seeded the vaginal mucosa after a lung infection with flu (104). In our study, the vast majority (~90%) of cells expressed CD49a across tissue types (4). Unfortunately, CD103 was not examined in that study. However, in studies of vaginal mucosa, the majority (>70%) expressed CD69 but not CD103 (CD49a was not measured in those studies) (104). Both studies support the idea that an antigen or repair is not strictly required to establish resident T cells, though it may affect their phenotype. What remains unresolved is which resident phenotypes are determined by priming in the SLO versus in the tissue during tissue-localized damage and repair. So-called “prime-pull” immunization strategies, using systemic priming and local tissue boosting with adjuvants, successfully generate T_RM_ in mucosal sites, including the lung and the female reproductive tract (109). It may be that optimal T_RM_ generation occurs when there is a combination of precursor instruction in the SLO and local antigen, inflammation, or tissue repair. In some ways, it makes sense to seed multiple tissues as a defense against future infections. The immune system doesn’t “know” where the next infection will occur. Not all influenza viruses, for example, are limited to the airways.

## Why is it important to know where T_RM_ come from?

T_RM_ contribute significantly to subsequent exposures to pathogens we have successfully become immune to. T cells with a T_RM_ phenotype are highly correlated with better prognoses in cancer. The T_RM_ phenotypes, defined by cell-surface expression of various markers, may be important because the markers confer functions crucial to protective immunity, not just because they are associated with a memory subset. A simple property, such as retention or motility in a tissue, may be sufficient to explain the protective advantages conferred. Due to their significance in infection and cancer, if we are to translate these into therapies and vaccination strategies, we need to understand how their functions and phenotypes are generated. Even the ability to identify and quantify relevant protective T cells is worthwhile. The basic ability to measure T_RM_ precursors in human blood during clinical trials, for example, is sorely lacking. Such a capability would be invaluable for evaluating and comparing alternative vaccination strategies aimed at generating resident memory. With this understanding and these tools, we could devise and compare rational approaches to improve human immunity.

The reliable generation of T_RM_ or T_RM_-like T cells *in vitro* remains largely untapped. A strategy for generating T_RM_-like CAR-T cells could be a powerful approach to treating solid tumors. While some essential signals that drive integrin expression have been identified, such as IL-12 and TGF-β, or the use of certain DCs (92, 93, 110, 111), and favorable metabolic conditions (81, 85–87, 112), there is no systematic protocol. The resulting cells are best described as T_RM_-like. Clearly, some signals/cues are missing. This is a general limitation of *in vitro* conditions. Most tissue culture media, for example, are deliberately enriched in glucose to promote survival and proliferation, while most tissues are glucose-poor and are instead rich in lipids and fatty acids. Methods for CAR-T generation emphasize cell expansion to very large numbers for adoptive transfer, which often relies on glucose-based metabolism. This is partly due to the inefficiency of cells reaching and remaining in peripheral sites where cancer cells reside. The physical context is also likely to influence outcomes, with access to matrix ligands and stromal cell components difficult to replicate in artificial settings. It is important to remember that the expression of these integrins or any marker is not synonymous with tissue residency, although they do correlate (though correlation is not causation)!. Regardless, integrins confer functions. Integrins regulate motility and may play a crucial role in positioning cells within the tissue, thereby bringing them into proximity to their ligands. To improve immunity, it may be sufficient that integrin-expressing TIL and T_RM_ are the most motile and tend to have stronger effector functions.

## The more we learn, the less we know (explaining heterogeneity)

While much is known about T_RM_ after nearly 30 years of study (**Box 1**), several questions of when and how T_RM_ originate remain open. Many observations of T_RM_ biology and development in the literature have relied on limited markers (especially CD69 and CD103). For instance, the concepts underlying the systemic (lymphoid) and local (tissue) models of T_RM_ development focus on the appearance and accumulation of CD103-expressing T cells (84, 92, 93), with less emphasis on other features. Clearly, resident memory is more heterogeneous, leaving gaps in our understanding of their biology. To wit, during an acute primary flu infection, a significant fraction of virus-specific CD8 T cells in the draining lymph node and spleen express CD49a as soon as they have expanded enough to be detected using MHC tetramers for the endogenous response (around day 5). If some of these are destined to become resident memory, it would support a lymphoid differentiation model. Could the models further evolve if other features (such as ICOS) were used? This suggests that the data used to support the models and the cellular features used to identify and trace cell fates need to be revisited. Our understanding is further limited because many T_RM_ studies sample only at early and late time points (or sometimes only late), after the T_RM_ have already formed and equilibrated. Events during the establishment phases are less well characterized. Clearly, more studies are needed to track precursor populations from priming (or before) to memory.

**Box 1 | Established observations regarding tissue-resident memory CD8 T cell development:**

- TGF-β derived from a variety of cellular and tissue sources is essential for TRM development (54, 62, 76, 96, 99, 113)
- TGF-β can induce both CD103 and CD49a integrins (48)
- IL12 promotes CD49a expression but suppresses CD103 (48)
- Developing CD8 T cells express CD103 in the thymus (68, 69)
- Migratory DC expressing TGF-β on the cell surface can induce epigenetic modifications to TRM-associated genes in otherwise naïve T cells and CD103 expression (96)
- Inhibition of DNGR1+ DC impairs T_RM_ formation (93)
- There is evidence of both TCR-dependent and independent bias in cells that become T_RM_ compared to circulating memory cells (21, 84, 103–105)

The current models are largely based on the location of resident memory differentiation, lymphoid versus peripheral. Neither accounts for the heterogeneity of T_RM_ within and between tissues. It is possible there is more than one pathway to residency. It is also possible that residency is not a terminal fate. In fact, it is already known that T_RM_ can become activated, expand, and differentiate into effectors and additional memory cells. Cellular immunologists are fond of ascribing terminal fates and names to T cell subsets (Th1, Th2, Th17, T_FH_, terminal effectors), and many of these are associated with developmental programs and the expression of specific transcription factors. The field has struggled to identify the “signature” genes of T_RM_. It may be elusive because their state is not fixed and multiple pathways exist. Clearly, the tissue in which they form influences their phenotypes and gene expression profiles, which allows them to adapt to their environment. It seems likely that some T_RM_ develop from continuous signals that begin shortly after activation in the lymphoid tissues and continue to receive further instructions, including tissue adaptation. This does not preclude that some T_RM_ develop from effectors in peripheral tissues, receiving their first instructions to become resident. T_RM_ precursors that find themselves in healthy tissues without active repair processes would have only received lymphatic cues and limited tissue-specific instructions. A given tissue would then have populations of resident memory T cells from at least three broad pathways (lymphoid-only, tissue-only, and both), suggesting that differentiation can be both discrete and continuous, with the tissue-adaptive features layered on top. Such a scenario would account for the observed heterogeneity and possibly explain the complex associations between T_RM_ features of TIL and clinical outcomes.

## Job security (work remaining)

Memory CD8 T cells that can reside in peripheral tissues (aka Tissue-Resident Memory, T_RM_) are an important first line of defense against antigenically drifted pathogens and damaged or mutated cells with the potential to become malignant. As much as our understanding of their biology has advanced, more remains to be learned. Firstly, there is substantial debate regarding the origins and necessary features of resident memory CD8 T cells. Although no markers are perfect, it has emerged that a relatively reliable surface protein expressed is the integrin CD49a. This protein is functionally important to T_RM_ biology, yet it remains to formally demonstrate that the CD49a-expressing T cells from early in the acute phase are, in fact, precursors of long-lived resident memory. This requires experimental tools to trace the fate of the early integrin-expressing T cells. Questions related to generating precursors in the SLO include determining why so few cells remain resident in the lymphoid tissues themselves. Are there regulatory mechanisms that ensure initial egress and/or limit lymphatic residency? It would also be useful to selectively antagonize integrin expression and functions at different times to probe their functional windows. Related to this, experiments are needed that show whether or not the same modes of specialized DC shown to prime CD103-expressing T_RM_ also hold true for the CD49a-expressing cells. These markers and transcription factors also imbue T_RM_ with unique survival and migration capabilities that could be exploited to improve the function of adoptive cellular immunotherapies. To do this, we need to develop knowledge and protocols to deliberately confer these features. Having more T_RM_ in the periphery is associated with improved clinical outcomes from infections. Mucosal vaccines that drive the generation and maintenance of resident memory T cells are a worthy goal, but to efficiently develop and evaluate different vaccination strategies, we need assays to measure these cells or their precursors in easy(er) to access peripheral blood samples. Investing in and developing novel vaccines is nigh impossible without reliable, inexpensive ways to measure successful outcomes. The field needs to advance further to realize the potential to translate T_RM_ biology into actionable interventions.
